# A second-sphere variant of cytochrome P450 PbdA improves turnover and apparent coupling in veratrate oxidation

**DOI:** 10.1186/s40643-026-01130-4

**Published:** 2026-09-10

**Authors:** Haiyan Song, Zeyang Li, Andong Li, Xinyu Cui, Shangxian Xie, Zhiguang Zhu

**Affiliations:** 1 Department of Biotechnology, Key Laboratory of Molecular Biophysics of the Ministry of Education, College of Life Science and Technology, Huazhong University of Science and Technology, Wuhan, 430074 China; 2 State Key Laboratory of Engineering Biology for Low-carbon Manufacturing, Tianjin Institute of Industrial Biotechnology, Chinese Academy of Sciences, 32 West 7th Avenue, Tianjin Airport Economic Area, Tianjin, 300308 China; 3 University of Chinese Academy of Sciences, 19A Yuquan Road, Shijingshan District, Beijing, 100049 China

**Keywords:** P450 PbdA, Second-sphere engineering, Apparent coupling efficiency, Active-site hydration, Cascade biocatalysis

## Abstract

Efficient use of reducing equivalents is critical for productive turnover in cytochrome P450 redox enzymes, yet improving activity without compromising substrate affinity remains challenging. Here, we engineered the second-sphere region of PbdA, a cytochrome P450 from *Rhodococcus jostii* RHA1 that catalyzes veratrate oxidation, and identified position 242 as a functionally sensitive site. The hydrophobic D242V variant approximately doubled the catalytic turnover number, *k*_cat_, while largely preserving substrate binding affinity. By quantifying NADH consumption and product formation, we found that D242V increased apparent coupling efficiency from 43.88% to 57.30%. Molecular dynamics simulations suggested that V242 remodels active-site hydration, reducing local water occupancy, which may help limit nonproductive uncoupling. Pathways analysis further revealed modest differences in the sampling of putative electron-transfer geometries, which correlated with the observed kinetic improvement but should be interpreted as a relative structural descriptor rather than direct evidence for altered electron-transfer rates. Finally, coupling engineered PbdA with glyoxylate carboligase enabled in situ formaldehyde scavenging and glycolaldehyde synthesis, achieving approximately 90% substrate conversion and 70% glycolaldehyde yield. These results suggest that while the second-sphere generally accommodates a highly restrictive mutational landscape, precise substitutions at isolated sensitive nodes (such as position 242) can occasionally fine-tune turnover and apparent coupling in P450-catalyzed veratrate oxidation, providing a basis for related biocatalytic cascades.

**Graphical abstract:**

**Figure:**
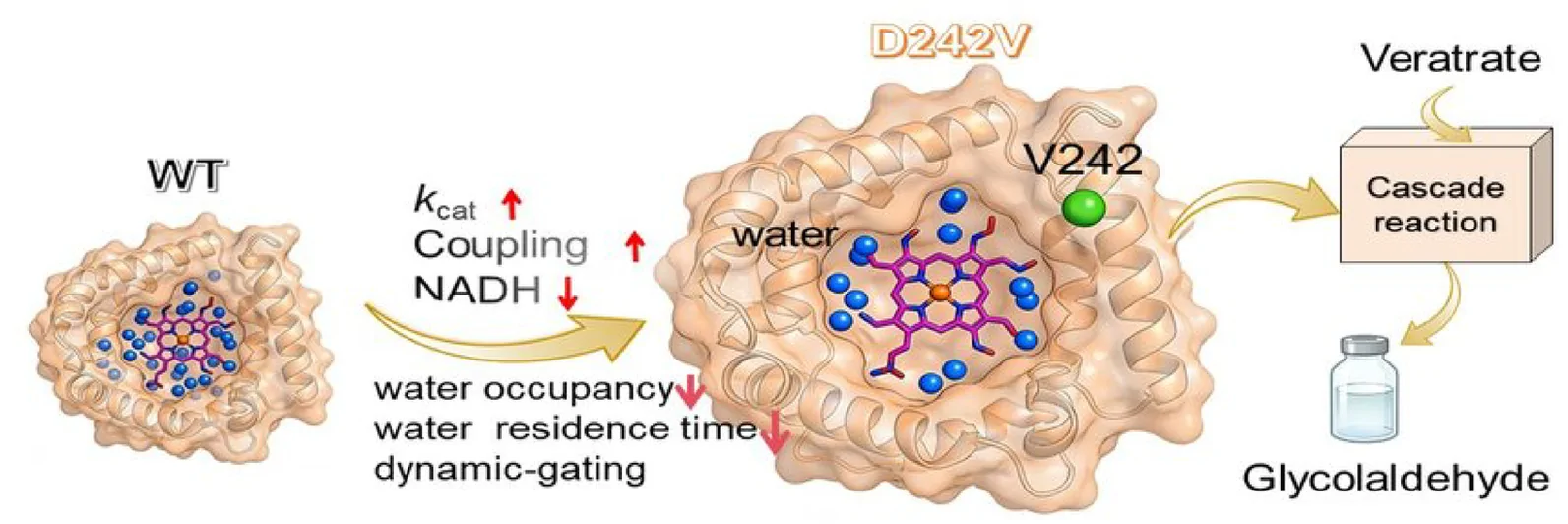

**Supplementary Information:**

The online version contains supplementary material available at https://doi.org/10.1186/s40643-026-01130-4.

## Introduction

Cytochrome P450 monooxygenases (P450s) are a highly versatile enzyme superfamily capable of functionalizing inert chemical bonds (Urlacher and Eiben 2006; Tang et al. 2026; Zhou et al. 2025; Zhao et al. 2024). In the context of a circular bioeconomy, P450s hold considerable promise for the *O*-demethylation of highly methoxylated lignin-derived aromatics into valuable platform chemicals (Wolf et al. 2024; Calabrese et al. 2026; Harlington et al. 2025; Li et al. 2025). PbdA, recently identified and characterized from *Rhodococcus jostii* RHA1, efficiently catalyzes the *O*-demethylation of *p*-methoxybenzoate (*p*-MBA) and related substrates such as veratrate (Wolf et al. 2024). Fundamentally, the catalytic cycle of this enzyme relies on precise, sequential delivery of two electrons from a reductase to the heme center (Deng et al. 2025a; Sugishima et al. 2014; Xu et al. 2025). However, the oxidative demethylation of aromatic methoxy groups remains kinetically demanding. Consequently, even for native or near-native substrates in vitro, kinetic mismatches among substrate binding, oxygen activation, proton delivery, and electron transfer can promote uncoupling (Ellis et al. 2021; Albertolle and Peter Guengerich 2018), which can divert reducing equivalents toward peroxide, superoxide, or water-forming uncoupling pathways, decreasing cofactor economy and potentially accelerating enzyme deactivation (Hamdane et al. 2008).

To overcome the critical kinetic barrier, rational structural engineering has been widely employed (Ellis et al. 2021; Deng et al. 2025a; Zhao et al. 2023). However, mutations of direct-contact residues, defined here as first-sphere active-site residues whose side chains directly interact with the bound substrate or reactive center, can disrupt finely tuned catalytic networks, inadvertently compromising essential substrate affinities (Meng et al. 2021; Denisov et al. 2005). Conversely, modifications in the third-sphere or more distal positions (> 10 Å) are generally considered less optimal for exerting direct, precise control over local active-site hydration dynamics. To mitigate these spatial limitations, expanding the engineering scope beyond direct-contact residues to the second coordination sphere has emerged as a promising strategy (de Visser 2020; Chaturvedi et al. 2023). The “second sphere” is operationally defined as residues located within ~ 5–10 Å of the substrate/reactive center that do not necessarily contact the bound ligand, but can influence access tunnels, local hydration, and conformational dynamics (Meng et al. 2022; Oprea et al. 1997; Bhatt and Dubey 2026). Because P450 uncoupling is often associated with excessive water access and fluctuating active-site water networks, targeted modifications in this region may reshape solvent connectivity and restrict non-essential bulk-water diffusion without perturbing the first-sphere catalytic core (Oprea et al. 1997; Deng et al. 2025b). Therefore, carefully tuning second-sphere interactions may reduce water-assisted off-pathway uncoupling while preserving native substrate affinity.

Despite advancements in intrinsic enzymatic kinetics, robust macroscopic bioconversion remains hindered by system-level limitations and byproduct toxicity (Intasian et al. 2021). Specifically, *O*-demethylation reactions stoichiometrically release highly reactive formaldehyde (Calabrese et al. 2026; Bleem et al. 2024; Pontel et al. 2015). Accumulation of this toxic C1 species can provoke kinetic stalling and nonspecific biocatalyst cross-linking, thereby limiting conversion (Jia et al. 2024).

In this work, we present a synergistic engineering framework to improve the catalytic efficiency of a model PbdA (PDB: 9G9S) (Wolf et al. 2024). Through structurally guided second-sphere engineering within the ~ 5–10 Å topological region, we identified a beneficial non-contact substitution, D242V, that nearly doubles *k*_cat_ while preserving substrate binding affinity. MD and pathway analyses are used to evaluate how second-sphere substitutions modulate hydration and conformational sampling relevant to productive turnover. To overcome the macroscopic accumulation barrier, we further couple the engineered P450 with a glyoxylate carboligase. The multi-enzyme cascade system is designed to continuously scavenge inhibitory formaldehyde, thereby relieving formaldehyde-associated inhibition and improving net conversion while upcycling the toxic C1 intermediate into glycolaldehyde, a high-value C2 building block.

## Experimental section

### Chemicals and materials

Disodium hydrogen phosphate, sodium dihydrogen phosphate, and sodium chloride (NaCl) were obtained from Sinopharm Chemical Reagent Co., Ltd. (China). The formaldehyde assay kit (acetylacetone colorimetric method) was purchased from Tianjin Baitaike Laboratory Equipment Sales Center (China). Tryptone and yeast extract were supplied by Oxoid Ltd. (UK). Antibiotics, including kanamycin and ampicillin, were acquired from Sangon Biotech (China). Isopropyl β-D-1-thiogalactopyranoside (IPTG), 5-Aminolevulinic acid hydrochloride (5-ALA) and imidazole were purchased from Solarbio Science & Technology Co., Ltd. (China). Catalase was obtained from Beijing Hwrk Chemical Co., Ltd. (China), and Coomassie Brilliant Blue G-250 was provided by Huabei Reagent Tianjin Co., Ltd. (China). TieChui *E. coli* Lysis Buffer was purchased from ACE Biotechnology Co., Ltd. (China). 3-Methyl-2-benzothiazolinonehydrazone Hydrochloride (MBTH) was obtained from Shanghai Yuanye Bio-Technology Co., Ltd. (China). The formaldehyde detection reagent was purchased from Wokai (China).

For molecular cloning, PrimeSTAR HS DNA polymerase was obtained from Takara Bio Inc. (Japan), and the restriction enzyme DpnI was sourced from New England Biolabs (USA). The ClonExpress II One Step Cloning Kit was purchased from Vazyme Biotech Co., Ltd. (China), and the Universal DNA Purification Kit was provided by Tiangen Biotech Co., Ltd. (China). *Escherichia coli* (*E. coli*) strains BL21(DE3) and Top10 were supplied by Biomed Gene Technology Co., Ltd. (China). All other chemicals used in this study were of analytical grade, and all solutions were prepared using ultrapure water (18.2 MΩ·cm^-1^) from a Milli-Q system.

### Selection of target residues for mutagenesis

Structure-based spatial analysis was performed utilizing the PyMOL molecular visualization system, using the crystal structure of the PbdA-veratrate complex (PDB ID: 9G9S) as the template. To systematically target the second-sphere topological region, a custom PyMOL script was employed to select amino acid residues located strictly within a 5–7 Å radius around the substrate, veratrate. Subsequently, the candidate residues within this defined shell were evaluated and filtered based on their physicochemical properties and potential functional roles in modulating the active site microenvironment, including polarity, hydrophobicity, electrostatic charge, and aromaticity. Ultimately, the following 13 s-sphere positions were rationally identified as key targets for site-saturation mutagenesis (SSM): 81, 84, 87, 88, 169, 171, 176, 234, 238, 239, 242, 243, and 289.

### Plasmid construction and site-saturation mutagenesis

The wild-type pbdA gene from *Rhodococcus jostii* RHA1 (corresponding to PDB: 9G9S) was cloned into the pET-28a(+) vector to construct the recombinant expression plasmid. Site-saturation mutagenesis was subsequently performed using a modified QuikChange protocol, utilizing this recombinant plasmid (pET-28a(+)-pbdA) as a template to introduce desired codon substitutions at the targeted positions. The mutagenesis was carried out in a 50 µL PCR reaction mixture containing 25 µL of PrimeSTAR Max Premix, 20 ng of the template plasmid, and 500 nM each of the forward and reverse mutagenic primers (Supplementary Table 1). The thermal cycling conditions were set as follows: an initial denaturation at 98 °C for 2 min; followed by 30 cycles of denaturation at 98 °C for 20 s, annealing at 60 °C for 20 s, and extension at 72 °C for 2 min; and a final extension at 72 °C for 10 min. Following amplification, the PCR products were treated with 20 U DpnI at 37 °C for 1 h to specifically degrade the methylated, non-mutated parental DNA templates. The linearized mutagenized fragments were then purified using a Universal DNA Purification Kit and subsequently circularized at 37 °C for 30 min using the ClonExpress II One Step Cloning Kit. The assembled ligation products were transformed into 100 µL of chemically competent *E. coli* TOP10 cells via heat shock at 42 °C. The transformed cells were recovered in an antibiotic-free Luria-Bertani (LB) medium at 37 °C for 1 h, then plated onto LB agar supplemented with 100 mg L^-1^ kanamycin, and incubated overnight at 37 °C. Single colonies were selected, and the successful incorporation of the target mutations was confirmed through Sanger sequencing. Finally, the sequence-verified variant plasmids were transformed into *E. coli* BL21(DE3) competent cells for subsequent heterologous protein expression.

### High-throughput screening procedure for PbdA variants

Following transformation into *E. coli* BL21(DE3), single colonies of the variants were inoculated into 96-well deep-well plates. Each well contained 0.5 mL of Terrific Broth (TB) medium supplemented with 100 mg L^-1^ kanamycin. To serve as positive and negative controls, respectively, the wild-type strain and the strain containing the empty expression vector were inoculated at the four corner wells of the plates. The pre-cultures were incubated overnight at 37 °C with shaking at 800 rpm. Subsequently, to provide sufficient aeration and volume for expression, the cultures were scaled up by transferring 100 µL of the overnight seed cultures into four 24-well deep-well plates (configured to emulate a 96-well layout) containing 5 mL of fresh TB medium with 100 mg L^-1^ kanamycin per well. After incubating at 37 °C and 800 rpm for 8 h, protein expression was induced by supplementing the cultures with 0.1 g L^-1^ 5-ALA and 0.2 mM IPTG. The plates were then continuously incubated overnight at 600 rpm.

The next day, cells were harvested by centrifugation. The obtained cell pellets were resuspended in 300 µL of TieChui *E. coli Lysis* Buffer and incubated at room temperature for 20 min to ensure complete cell lysis. Cell debris was removed by centrifugation, and the resulting clear cell-free supernatant was collected for the subsequent catalytic reaction. The enzymatic assay was performed in a 200 µL reaction system consisting of 20 mM PBS, 2 mM NADH, 1 µM putidaredoxin (Pdx), 1 µM putidaredoxin reductase (Pdr), 1 mM veratrate as the substrate, 100 U catalase, and 100 µL of the cell-free supernatant. The reaction mixtures were incubated at 25 ℃ for 30 min. After removing any precipitates by brief centrifugation, the evaluation of the variant activity was carried out by quantifying the released formaldehyde. Briefly, 100 µL of the reaction solution was transferred into a standard 96-well microtiter plate and mixed with 100 µL of acetylacetone-based formaldehyde detection reagent, also known as Nash reagent. In this colorimetric assay, formaldehyde reacts with acetylacetone in the presence of ammonium acetate to form a yellow diacetyldihydrolutidine derivative, which can be monitored at approximately 420 nm (Nash 1953). The initial absorbance at 420 nm was measured immediately. After allowing the color to develop for 2 h at room temperature, the terminal absorbance at 420 nm was recorded. The relative catalytic efficacy of the PbdA variants was subsequently estimated by calculating the difference in absorbance between the two time points.

### Expression and purification of PbdA, Pdx, Pdr and GCL

The expression and purification of PbdA, Pdx, Pdr and GCL were as follows: fifty microliters of the glycerol stocks for the strains expressing PbdA, Pdx, Pdr and GCL were individually inoculated into 10 mL of Luria-Bertani (LB) medium supplemented with appropriate antibiotics. These seed cultures were incubated overnight at 37 °C with shaking at 220 rpm. Then the pre-cultures were directly transferred into 1 L of fresh LB medium containing the corresponding antibiotics. The cultures were grown at 37 °C until the optical density at 600 nm reached 0.6-1.0. Subsequently, protein expression was induced. For the expression of PbdA, the culture was supplemented with 0.1 g L^-1^ 5-ALA and 0.2 mM IPTG. For Pdx, Pdr and GCL expression was induced by the sole addition of 0.2 mM IPTG. All cultures were continuously incubated overnight for protein expression. Only Pdr carries ampicillin resistance, while all others carry kanamycin resistance. The final concentration for all antibiotics was 100 mg L^-1^.

Cells were harvested by centrifugation at 6000 rpm for 10 min. The obtained cell pellets were washed with 20 mM phosphate-buffered saline (PBS) and then completely resuspended in the same buffer. Cell disruption was performed using a high-pressure homogenizer. Following cell lysis, the lysates were centrifuged at 8000 rpm for 30 min to remove cell debris, and the resulting clear cell-free supernatants were collected.

The target proteins were purified via Ni-affinity chromatography. Briefly, the collected supernatant was loaded onto a Ni-NTA column. Nonspecific binding proteins were washed away with a buffer containing 20 mM imidazole, and the target His-tagged proteins were subsequently eluted using a buffer containing 200 mM imidazole. The eluates were then subjected to ultrafiltration to remove the imidazole and exchange the buffer. 2 mM DTT was added into Pdx. Finally, the purified proteins were aliquoted and stored at − 20 °C for future use.

All the source organisms, sequences, and accession numbers of the enzymes used in this study were in Supplementary Table 2.

### Determination of PbdA concentration

The concentration of PbdA was determined using the classic pyridine hemochromogen assay. Briefly, two working solutions were prepared: Solution A contained 0.2 M NaOH, 40% (v/v) pyridine, and 500 µM potassium ferricyanide; Solution B consisted of 0.5 M sodium dithionite dissolved in 0.5 M NaOH. For the measurement, a 500 µL aliquot of the PbdA sample was mixed with 500 µL of Solution A, followed by the addition of 10 µL of Solution B. The absorbance of the final mixture was continuously monitored at 557 nm until a stable reading was achieved. The exact concentration of PbdA was then calculated using a molar extinction coefficient (ε557) of 34.7 mM^-1^ cm^-1^ (Barr and Guo 2015; Berry and Trumpower 1987).

### Substrate binding assay

The substrate binding assay was performed at room temperature using a Miou ND-100 C UV-Vis spectrophotometer (China) equipped with a 1 cm path-length quartz cuvette. Titration was conducted by sequentially adding small aliquots of veratrate, and the corresponding absorption spectra were recorded after each addition. Upon substrate binding, a characteristic Type I spectral shift was observed, characterized by the displacement of the Soret band from 420 nm to 390 nm. Finally, the dissociation constant (*K*_d_) was determined by plotting the absorbance differences (Δ390–Δ420 nm) against the substrate concentrations and fitting the data using GraphPad Prism software.

### Kinetic characterization

Steady-state kinetic assays were performed in a total reaction volume of 200 µL. The standard reaction mixture comprised 1 µM PbdA, 10 µM Pdx, 1 µM Pdr, 100 U catalase, 2 mM NADH, and varying concentrations of the substrate. Following incubation at room temperature for 10 min, the reactions were quenched by the addition of sulfuric acid. The mixtures were subsequently centrifuged at 12,000 rpm to remove precipitated proteins, and the resulting supernatants were collected for product quantification via high-performance liquid chromatography (HPLC). The apparent kinetic parameters (*K*_m_ and *k*_cat_) were determined by fitting the initial velocity data to the Michaelis-Menten equation using non-linear regression analysis in GraphPad Prism software. Specifically, the apparent *k*_cat_ was calculated using the equation *k*_cat_=*v*_max_/[*E*]_t_, where *v*_max_ is the fitted maximum reaction rate (mM⋅min^-1^) and [*E*]_t_ is the final concentration of PbdA (1 µM) in the reaction.

### Determination of apparent coupling efficiency

To determine the apparent coupling efficiency, the reaction was performed in a total volume of 1 mL. The reaction mixture consisted of 1 µM PbdA (wild-type or variants), 1 µM Pdr, 100 U catalase, 2 mM NADH, and 1 mM veratrate with different concentration of Pdx. To quantify NADH consumption, the decrease in absorbance was monitored at 340 nm. In parallel, the reaction was incubated for 30 min, after which the concentration of the product, vanillic acid, was determined by HPLC. Finally, the apparent coupling efficiency was calculated based on the ratio of vanillic acid generated to the amount of NADH consumed.

### Molecular dynamics (MD) simulation

The initial structures of the wild-type complex (PDB: 9G9S) and its D242V and D242S variant were prepared for molecular dynamics (MD) simulations using the AMBER 24 software package. The protein was parameterized with the ff19SB force field. Parameters for the metal center (Fe) and its coordinated atoms, including the bound heme and small molecules, were generated using the MCPB.py tool and the Generalized AMBER Force Field (GAFF), with partial charges derived appropriately via the AM1-BCC/RESP method. Explicit bonds were defined between the iron center and its coordinating atoms. The complexes were solvated in a cubic TIP3P water box containing approximately 15,510 water molecules with a 12 Å buffer distance. The systems were neutralized by adding 8 Na⁺ ions.

Prior to the production runs, the systems were subjected to energy minimization for 6,000 steps, consisting of 3,000 steps of the steepest descent method followed by 3,000 steps of the conjugate gradient method, while a harmonic restraint of 50.0 kcal/(mol·Å^2^) was applied to the heavy atoms of the protein and ligands. Subsequently, the systems were gradually heated from 0 to 300 K in the NVT ensemble using a Langevin thermostat with a collision frequency of 2.0 ps^-1^, during which weak positional restraints (10.0 kcal/(mol·Å^2^)) were applied. After appropriate equilibration in the NPT ensemble, a 200-ns production MD simulation was performed for each system.

During all simulations, the SHAKE algorithm was utilized to constrain bonds involving hydrogen atoms, allowing for a time step of 2 fs. Long-range electrostatic interactions were computed using the Particle Mesh Ewald (PME) algorithm, and the non-bonded cutoff distance was set to 10.0 Å. All MD simulations were accelerated using the pmemd.cuda engine on an NVIDIA GeForce RTX 4090 GPU. System coordinates were saved every 100 ps. Finally, trajectory analyses, including Root Mean Square Deviation (RMSD) and Root Mean Square Fluctuation (RMSF), were performed using the CPPTRAJ module of AmberTools.

To monitor the active site hydration dynamically, the radial distribution function (RDF) between the heme iron and water oxygen atoms was calculated using CPPTRAJ. The average RDF values within a 10 Å radius were then extracted as a function of simulation time. Furthermore, to evaluate the solvation changes at the active site, the total number of water molecules within a 10 Å radius of the heme center was calculated as a function of simulation time using CPPTRAJ.

Because these analyses are based on finite sampling, the MD results are interpreted as qualitative, hypothesis-generating trends; robust quantitative estimates would require additional independent trajectories and/or longer simulations.

### Vanillic acid and glycolaldehyde analysis by HPLC

The quantitative analysis of vanillic acid was performed using an HPLC system equipped with a C18 column. The separation was carried out under isocratic elution conditions. The mobile phase consisted of 0.1% formic acid in water (Solvent A) and acetonitrile (Solvent B) at a ratio of 80:20 (v/v). The flow rate was maintained at 1.0 mL min^-1^, and the column temperature was set to 30 °C. The detection wavelength was set at 272 nm, and the total run time for each injection was 30 min.

For the detection of glycolaldehyde, a 100 µL aliquot of the reaction mixture was derivatized with 100 µL of 10 mM MBTH at room temperature for 24 h. The mixture was then centrifuged at 12,000 rpm for 10 min, and the supernatant was highly collected for HPLC analysis. Chromatographic separation was performed using a C18 column maintained at 30 °C. The mobile phase consisted of 0.1% formic acid in water (Solvent A) and 100% acetonitrile (Solvent B). A 30 min gradient elution program was applied as follows: 20% B at 0–1.50 min, linearly increasing to 100% B at 15.00 min, held at 100% B until 20.00 min, returning to 20% B at 21.00 min, and maintained at 20% B until 30.00 min for column re-equilibration. The flow rate was maintained at 1.0 mL min^-1^ and the detection wavelength was set at 272 nm.

### GCL-coupled enzymatic reaction

For the GCL-coupled enzymatic reaction, the assay was performed in a total volume of 3 mL at 25 °C. The reaction mixture contained 1 µM PbdA (wild-type or variant), 2 g L^-1^ GCL, 10 µM Pdx, 1 µM Pdr, 100 U catalase, 2 mM NADH, 2 mM Mg^2+^, 2 mM TPP and 1 mM veratrate. During the reaction incubation, two 100 µL aliquots were collected every hour and immediately quenched with 10% sulfuric acid. The samples were then centrifuged at 12,000 rpm for 10 min. The resulting supernatants were analyzed as follows: one aliquot was directly used to quantify the concentration of vanillic acid, while the second aliquot was mixed with 100 µL of 10 mM MBTH and derivatized for 24 h for the detection of glycolaldehyde.

### Hydrogen peroxide detection

Hydrogen peroxide accumulation was measured using a colorimetric assay. The reaction mixture comprised 1 µM PbdA, 10 µM Pdx, 1 µM Pdr, 2 mM NADH, and 1 mM veratrate, with or without 100 U catalase. After incubation for 1 h, H_2_O_2_ was detected by adding 10 U horseradish peroxidase (HRP), 1 mM 4-aminoantipyrine (4-AA), and 1 mM N-ethyl-N-(2-hydroxy-3-sulfopropyl)-3-methylaniline (TOOS). The resulting quinoneimine dye was quantified by measuring absorbance at 555 nm. Catalase-containing reactions were included to confirm the specificity of the H_2_O_2_-derived signal.

## Results and discussion

### Structure-guided engineering and library screening

**Fig. 1:**
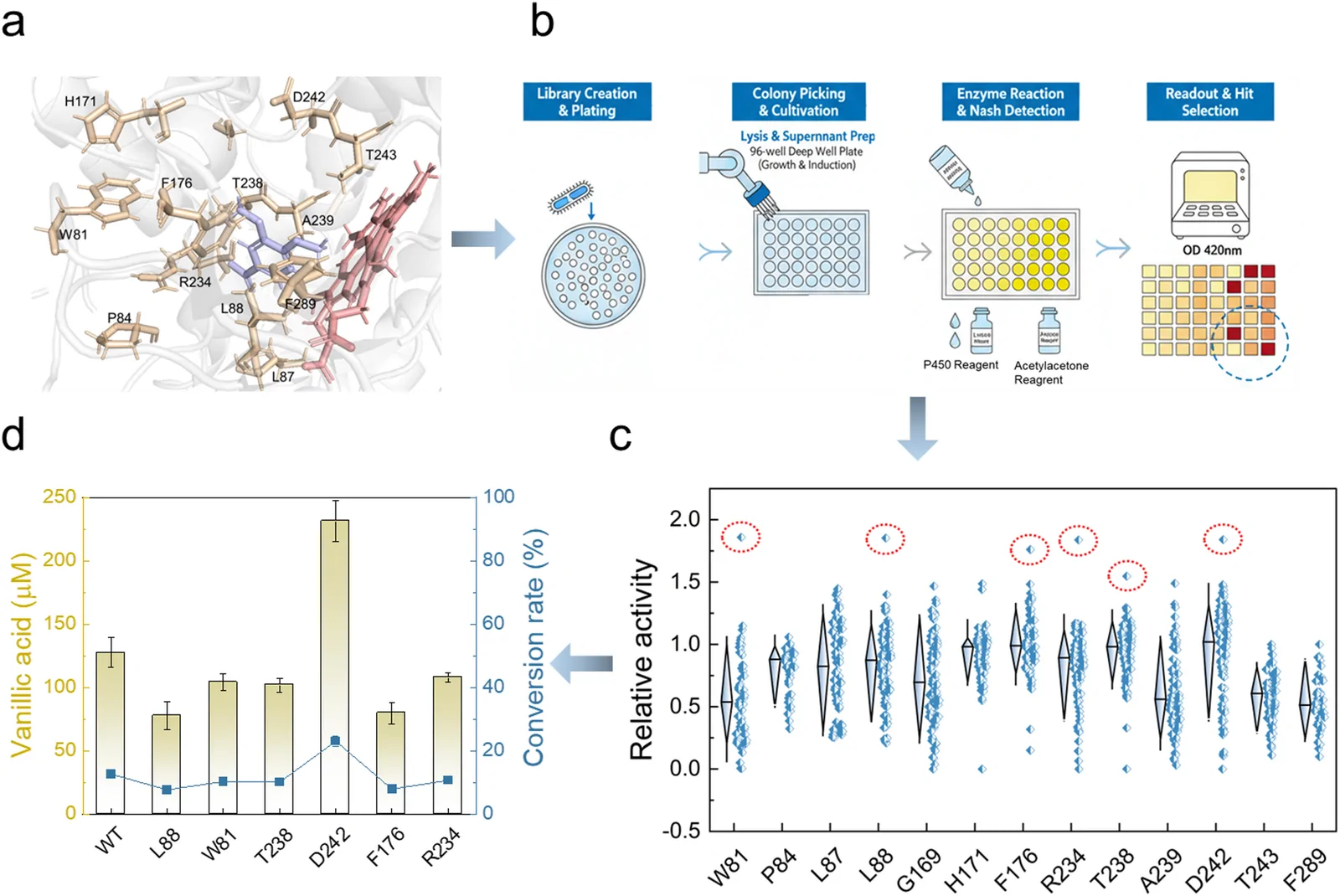
**a** Preselected variant sites mapped onto the structure (PDB:9G9S). Heme (HEM) is shown in red, the substrate veratrate in purple, and the candidate amino acids in yellow. **b** Schematic of high-throughput screening process. **c** Enzymatic activity assay results for the library measured in a 96-well plate format. The red dashed circle points out a well containing a variant with improved activity, which was selected from the 96-well plate for further rescreening. **d** Substrate conversion rates of initially selected variants identified via high-throughput screening. The standard reaction conditions were: 20 mM PBS (pH 7.0), 1 µM PbdA, 1 µM Pdx, 1 µM Pdr, 1 mM veratrate, 2 mM NADH, and 100 U catalase. The reaction time was 30 min. Added 100 U of catalase was used to remove H_2_O_2_ formed during uncoupling, thereby minimizing oxidative inactivation during the endpoint screening assay. Data are shown as mean ± SD (*n* = 3 independent experiments)

To engineer PbdA for enhanced catalytic performance, we employed a structure-guided semi-rational design strategy based on the crystal structure of the PbdA-veratrate complex (PDB: 9G9S). We focused on the second-sphere region surrounding the bound substrate, operationally defined as residues located within ~ 5–10 Å of the substrate/reactive center. In practice, candidate positions were prioritized from the inner portion of this shell (typically ~ 5–7 Å) and further filtered by physicochemical features (polarity, hydrophobicity, charge, and aromaticity) and by proximity to putative access tunnels and solvent networks. Consequently, 13 s-sphere positions were selected for site-saturation mutagenesis (SSM): 81, 84, 87, 88, 169, 171, 176, 234, 238, 239, 242, 243, and 289 (Fig. 1a). A mutant library was constructed and subjected to high-throughput screening in a 96-well plate format (Fig. 1b). Variants exhibiting activity > 1.5-fold relative to wild type (WT) in the primary screen were selected for rescreening (Fig. 1c). To minimize false positives arising from expression variability, hit variants were purified from shake-flask cultures and evaluated in vitro using NADH as the electron donor and putidaredoxin/putidaredoxin reductase (Pdx/Pdr) as redox partners. Reactions containing 1 mM veratrate were incubated for 30 min, and vanillic acid formation was quantified by HPLC (Fig. 1d and Supplementary Figs. 1 and 2). Among the purified hits, the D242 variant displayed approximately two-fold higher substrate conversion than WT (Fig. 1d), with clear depletion of veratrate accompanied by increased vanillic acid formation (Supplementary Fig. 3). DNA sequencing identified the beneficial substitution as D242V. Collectively, the structure-guided second-sphere screening identified D242V as a key variant with improved catalytic performance, motivating further mechanistic analysis and system-level optimization.

### Optimization of the biocatalytic system

**Fig. 2:**
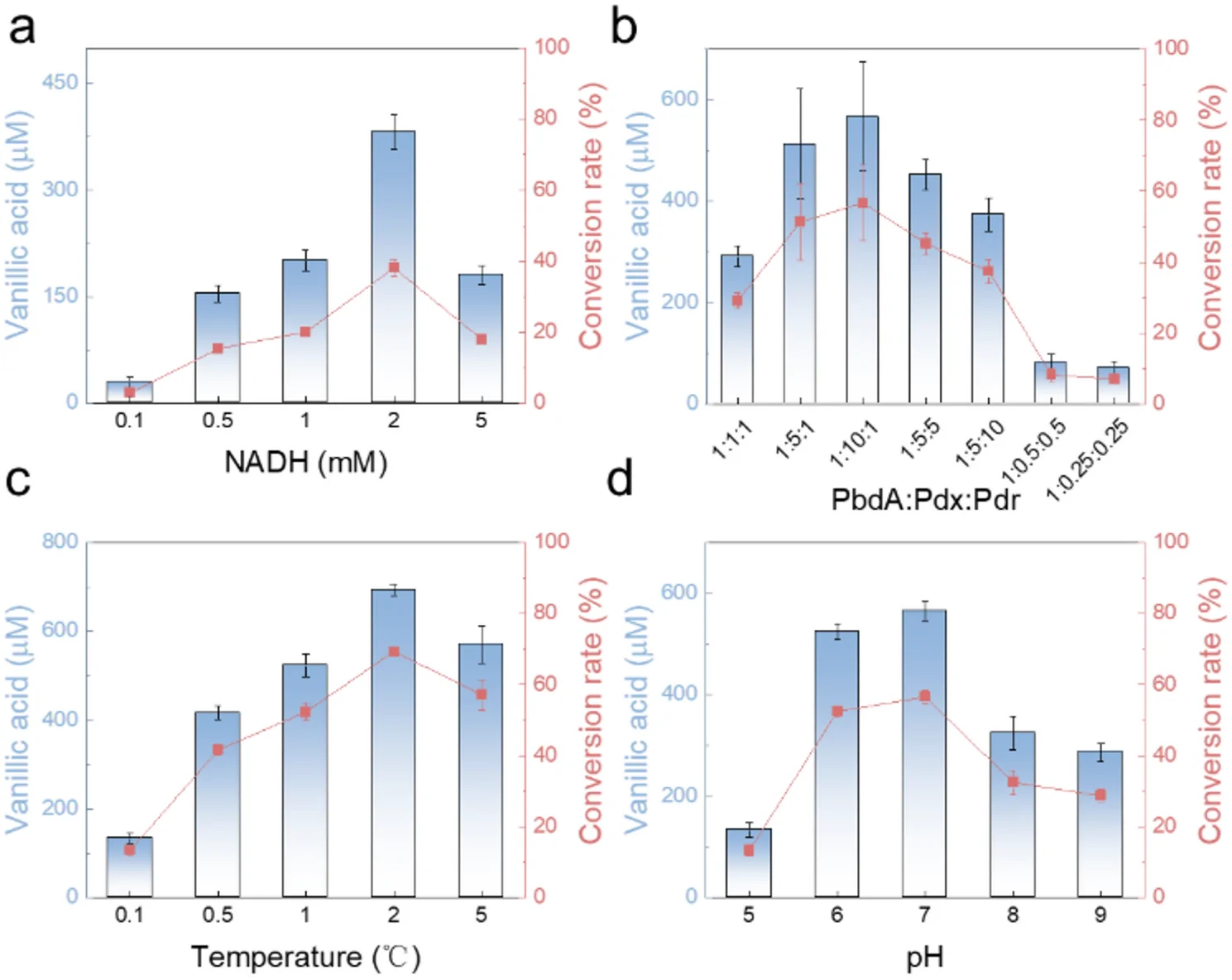
Optimization of the reconstituted biocatalytic system. **a** NADH concentration (0.1, 0.5, 1, 2, 5 mM). **b** PbdA: Pdx: Pdr molar ratio (1:1:1, 1:5:1, 1:10:1, 1:5:5, 1:5:10, 1:0.5:0.5, and 1:0.25:0.25). **c** Temperature (15–35 °C). **d** pH (5–9). Unless otherwise specified, reactions were performed in 20 mM PBS with 1 µM PbdA, 1 mM veratrate, and 100 U catalase for 30 min. Data are shown as mean ± SD (*n* = 3 independent experiments)

To translate the intrinsic advantages of D242V into improved apparent biocatalytic performance, we alleviated macroscopic bottlenecks by tuning redox stoichiometry and reaction conditions in the reconstituted P450 system. As shown in Fig. 2a, vanillic acid production exhibited an optimum at an intermediate NADH concentration of ~ 2 mM. At higher NADH loading, the apparent yield decreased, suggesting that an oversupply of NADH-derived reducing equivalents may impair overall system performance. Because overall flux depends on kinetic matching among PbdA, Pdx, and Pdr, we next optimized the redox-partner stoichiometry. The highest product titer was obtained at a PbdA: Pdx: Pdr molar ratio of 1:10:1 (Fig. 2b). The requirement for excess Pdx is consistent with its role as a diffusional electron shuttle, where higher Pdx concentrations are expected to increase the frequency of productive encounters and the probability of successful electron delivery to PbdA (Hirakawa and Nagamune 2010). Biochemical condition optimization further highlighted the fragility of the reconstituted components. The system performed best under mild conditions of 25 °C and pH 7.0 (Fig. 2c and d). Reduced activity at elevated temperature likely reflects compromised stability of the [2Fe-2 S] cluster in Pdx, while deviations from neutral pH can perturb redox potentials or protonation states important for dioxygen activation (Sligar et al. 2005). Together, these condition and stoichiometry optimizations increased the overall substrate conversion from ~ 20% to ~ 58% under the tested conditions. To determine whether the underperformance of other previously screened variants (Fig. 1d) could be rescued by alleviating these macroscopic bottlenecks, we re-evaluated WT, L88, W81, T238, and F176 under the newly established optimal conditions. As shown in Supplementary Fig. 4, their product yields and conversion rates remained lower than those of WT under the tested conditions, indicating that their biocatalytic limitations are intrinsic and further highlighting the distinct beneficial effect of the D242V substitution.

### Substrate affinity, steady-state kinetics and apparent coupling efficiency of WT, D242V and D242S

**Fig. 3:**
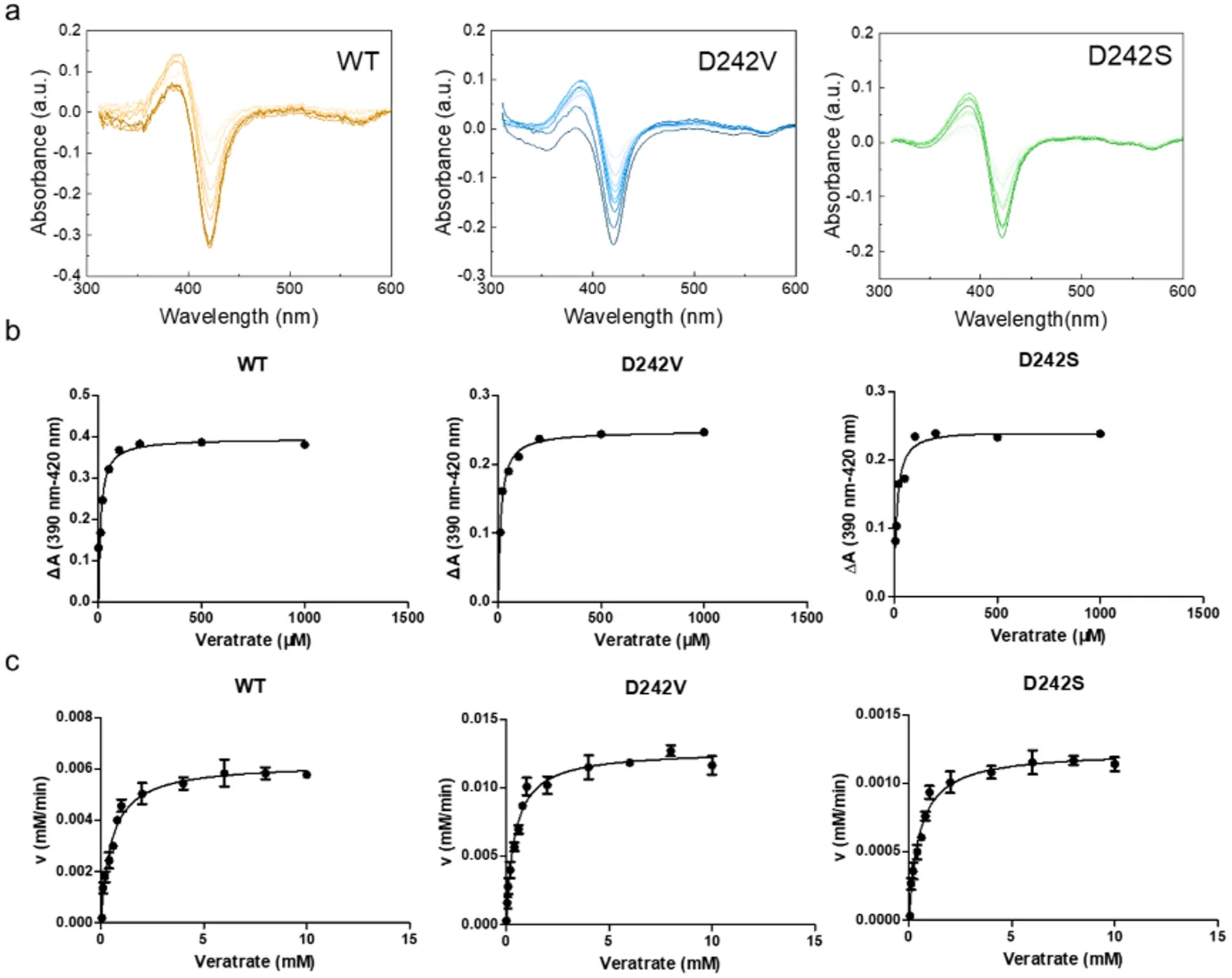
**a** Substrate binding of WT, D242V and D242S. Difference spectra of PbdA titrated with 1 µM to 1 mM veratrate from the substrate-free enzyme. Darker colors indicate higher concentration of veratrate. Spectra were collected after each addition of substrate to 3 µM WT and 2 µM D242V and D242S in 20 mM PBS pH 7.0. **b** Binding isotherm of WT, D242V and D242S with veratrate. The fitted curve represents a simple hyperbola. **c** Michaelis-Menten kinetics of the WT, D242V and D242S. Initial reaction velocities (*v*) were measured at varying substrate concentrations from 0.1 mM to 10 mM and fitted to the Michaelis-Menten equation. The standard reaction conditions were: 20 mM PBS (pH 7.0), 1 µM PbdA, 10 µM Pdx, 1 µM Pdr, 2 mM NADH, and 100 U catalase. The reaction time was 10 min

After identifying D242V as a beneficial mutation, we introduced the hydrophilic D242S variant as a counterpart to the hydrophobic Val substitution to further examine the role of residue 242. We then characterized the spin-state equilibrium and steady-state kinetics of WT, D242V, and D242S to determine whether the enhanced performance of D242V was associated with altered substrate recognition or catalytic turnover. As shown in Fig. 3a and Supplementary Fig. 5, the titration of the substrate induced a characteristic Type I spectral shift (absorbance decrease at ~ 418 nm and increase at ~ 390 nm) in WT, D242V, and D242S, indicating a successful displacement of the water ligand by the substrate(Podgorski et al. 2024). The calculated spectral dissociation constants (*K*_d_) were comparable among the WT and the variants (Fig. 3b; Table 1), suggesting that mutations at position 242 do not significantly alter the substrate binding affinity. We then examined the steady-state kinetic parameters (Fig. 3c; Table 1). The comparable spectral *K*_d_ values indicate similar substrate binding in the ferric resting state, whereas the similar apparent *K*_m_ values suggest that substrate availability is unlikely to be the dominant factor underlying the improved activity under the steady-state assay conditions. However, a striking difference was observed in the catalytic turnover number (*k*_cat_). The D242V variant exhibited a *k*_cat_ value nearly twice that of the WT. In contrast, substitution at this position with the structurally similar but hydrophilic Ser led to reduced catalytic activity. The D242S variant exhibited a drastically reduced *k*_cat_ of 1.24 min^-1^, which indicates that local hydrophobicity appears to be an important determinant of catalytic efficiency rather than structural geometry alone. Thus, under these assay conditions, the enhanced activity of D242V is most consistent with accelerated catalysis rather than strengthened substrate binding.

**Fig. 4:**
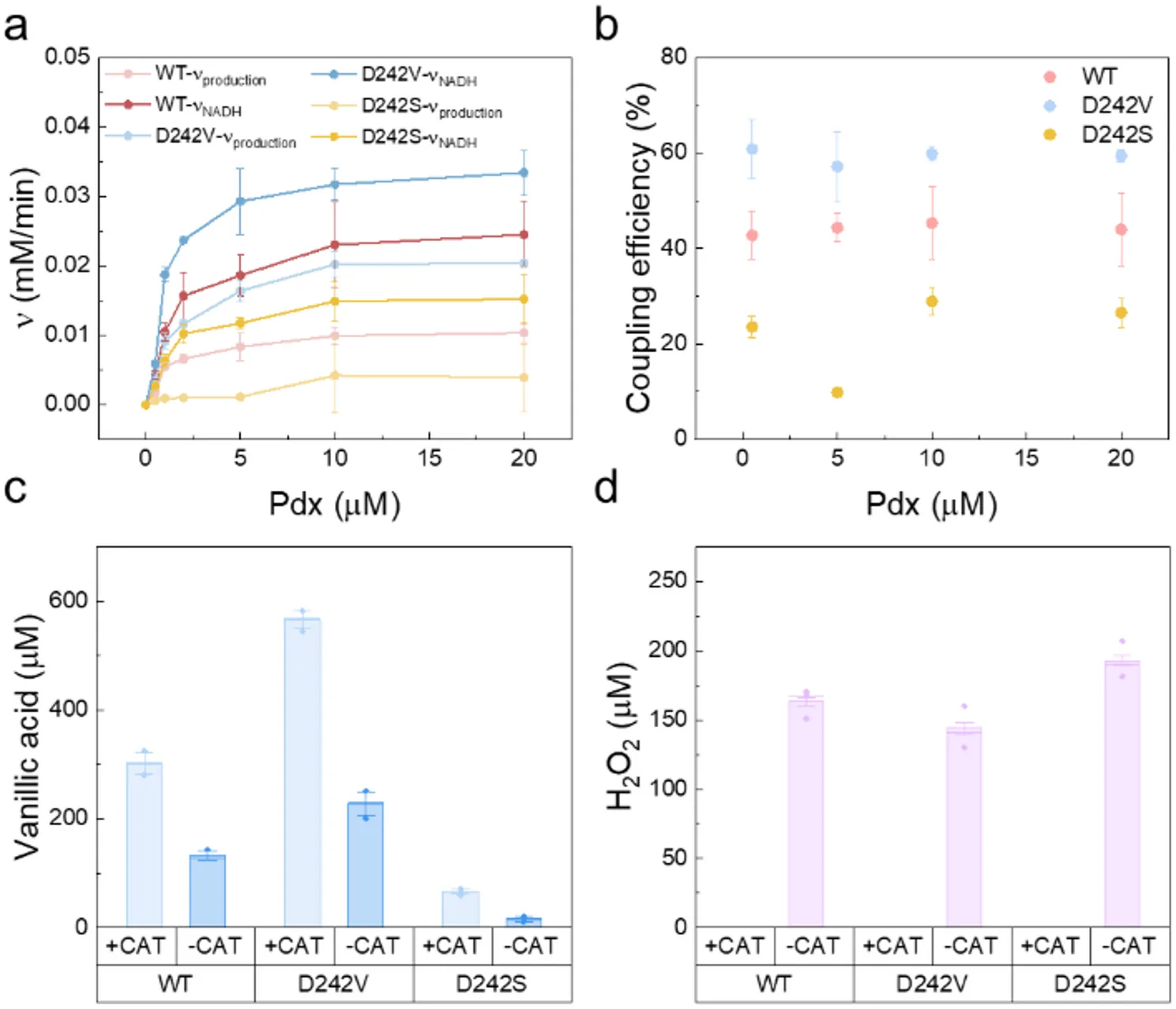
**a** The reaction rates of NADH consumption and product formation were measured at increasing Pdx concentrations for WT, D242V and D242S. **b** Apparent coupling efficiency of WT, D242V and D242S. Vanillic acid (**c**) and H_2_O_2_ (**d**) production of WT, D242V and D242S with and without catalase (100 U). Data are shown as mean ± SD (*n* = 3 independent experiments)

To evaluate whether the higher turnover is accompanied by more productive use of reducing equivalents, we quantified NADH consumption and product formation while titrating Pdx (Fig. 4a). Apparent coupling efficiency was operationally defined as 100 × *v*_product_/*v*_NADH_, where *v*_product_ is the rate of vanillic acid formation and *v*_NADH_ is the rate of NADH consumption. Because one NADH molecule provides the two reducing equivalents required for one productive P450 monooxygenation event, the value estimates the fraction of NADH oxidation that leads to product formation. At 10 µM Pdx, D242V increased apparent coupling efficiency from 43.88% to 57.30% (Fig. 4b; Table 1), corresponding to a reduction in NADH cost. Conversely, the hydrophilic D242S substitution showed a lower apparent coupling efficiency of 22.22% (Fig. 4b; Table 1). Catalase (CAT)-rescue assays showed that catalase increased vanillic acid formation for all enzymes, with D242V retaining the highest product formation, whereas D242S remained poorly active (Fig. 4c). In the absence of catalase, direct H_2_O_2_ quantification showed lower H_2_O_2_ accumulation for D242V than for WT, whereas D242S accumulated the highest H_2_O_2_ level. In the presence of catalase, H_2_O_2_ was below or close to the detection limit, consistent with efficient peroxide removal under these assay conditions (Fig. 4d). These data support reduced peroxide-forming uncoupling in D242V, although the apparent coupling metric reflects overall NADH partitioning and does not distinguish among peroxide-, superoxide-, and water-forming uncoupling pathways.

**Table 1:** Binding and kinetic parameters of WT, D242V and D242S

	K_d_ (µM)	%High-spin (200 µM veratrate)	K_M_ (mM)	k_cat_ (min^− 1^)	k_cat_/K_M_ (min^−1^mM^− 1^)	Apparent coupling efficiency (%)
WT	10.8 ± 3.8	97.9%	0.49 ± 0.06	6.21 ± 0.20	12.67 ± 1.60	43.88 ± 1.06%
D242V	12.3 ± 1.4	95.3%	0.41 ± 0.04	12.73 ± 0.82	31.05 ± 3.63	57.30 ± 4.76%
D242S	12.1 ± 2.8	95.7%	0.54 ± 0.05	1.24 ± 0.04	2.30 ± 0.23	22.22 ± 8.54%

### Analysis of molecular dynamics simulations

To gain mechanistic insight into the effects of the mutations, we performed 200 ns molecular dynamics (MD) simulations. Backbone RMSD analysis showed that all systems remained within a relatively stable range (Supplementary Fig. 6a). RMSF analysis further revealed modest increases in local flexibility for D242V in several loop regions, whereas D242S displayed more pronounced local fluctuations (Supplementary Fig. 6b). Considering that D242V showed substrate-binding affinity and high-spin population similar to WT but improved *k*_cat_ and apparent coupling efficiency, these simulations suggest that its enhanced activity may be associated with subtle changes in the active-site microenvironment rather than altered substrate binding. We therefore analyzed the hydration dynamics within the heme pocket.

**Fig. 5:**
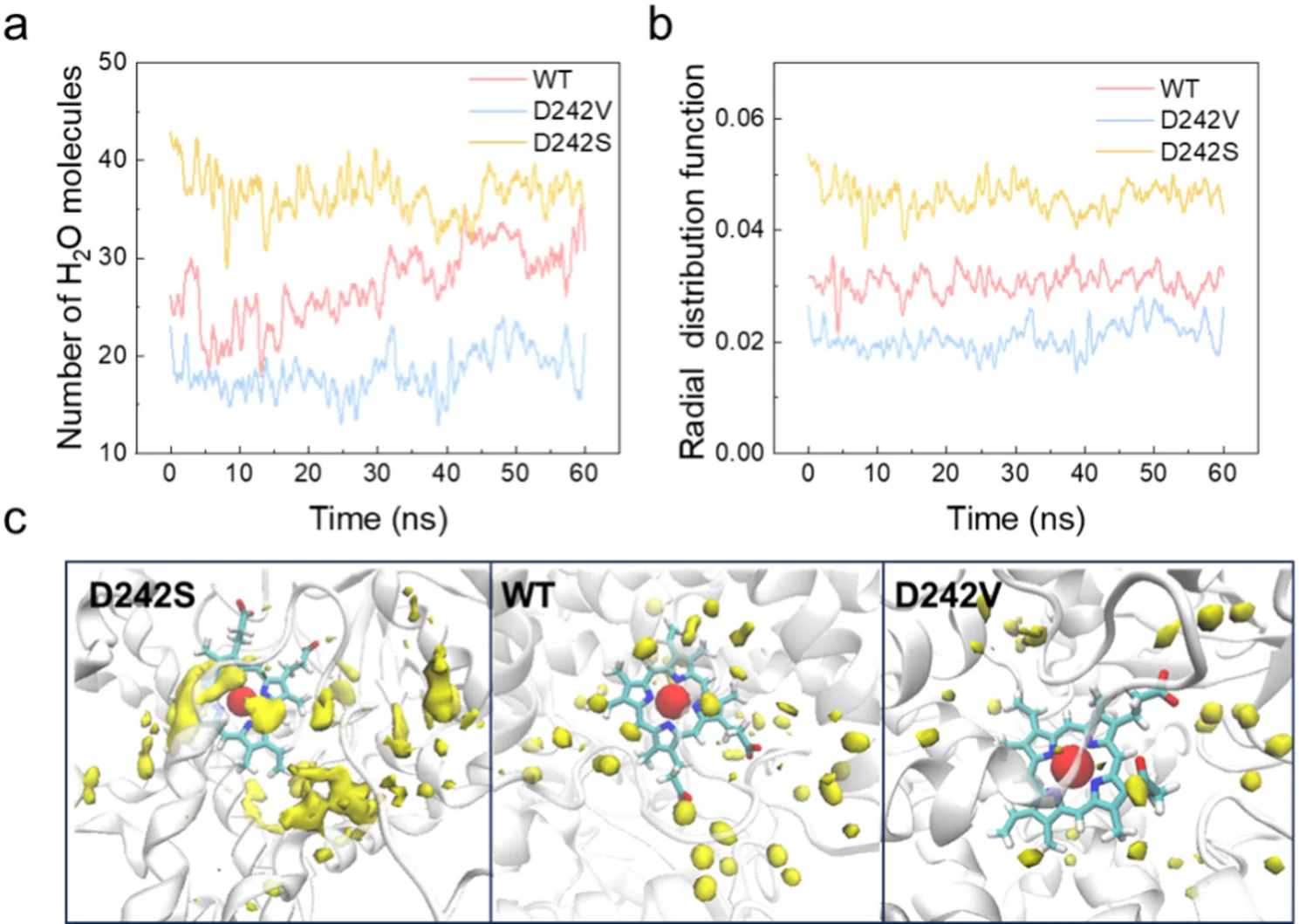
Hydrophobic desolvation and destabilization of the active site water network in WT, D242V and D242S. All analyses were performed on the equilibrated 60 ns trajectory window (corresponding to 120–180 ns of the total simulation). **a** Time evolution of the number of water molecules within 10 Å of the heme iron, showing a persistent reduction. **b** Time-dependent integrated RDF-derived hydration index within 10 Å of the heme center. **c** Spatial probability density maps for WT, D242V and D242S

To explore how these mutations may affect the active-site microenvironment, we further characterized the hydration dynamics within the heme pocket. As shown in Fig. 5a and Supplementary Fig. 7a, the time evolution of the number of water molecules reveals an overall desolvation effect in the D242V variant, whereas the D242S mutation leads to excessive water accumulation. The divergent local hydration is corroborated by the radial distribution function (RDF) of water oxygen atoms relative to the heme center, which shows lower density for D242V and higher density for D242S (Fig. 5b and Supplementary Fig. 7b). Spatial distribution maps of water occupancy further visualize this phenomenon (Fig. 5c). The WT active site displays a densely populated continuous water network, whereas the spatial water density is decreased in D242V but increased in D242S. Additional residence-time analyses showed a modest tendency toward shorter water retention in D242V, further supporting reduced active-site hydration dynamics, although the effect was less pronounced than the occupancy differences (Supplementary Fig. 8). These analyses collectively suggest that active-site water molecules in D242V are less abundant and, to a lesser extent, more transient. We propose that the hydration remodeling in D242V may reduce the probability of water-assisted off-pathway events by limiting the persistence of bulk-like water networks near reactive intermediates, and is consistent with the experimentally observed increase in apparent coupling efficiency (Meng et al. 2022), whereas the flooded active site of D242S may exacerbate non-productive uncoupling.

The comparable *K*_d_, high-spin populations, and *K*_M_ values of WT, D242V, and D242S indicate that the divergent catalytic behaviors are unlikely to arise primarily from altered substrate binding or spin-state conversion. While our hydration analysis (Fig. 5) clearly points to a water-exclusion mechanism for improved coupling, we also exploratorily examined whether the mutations might affect putative post-binding coupling geometries. Using the VMD Pathways plugin as a purely geometry-based heuristic tool, we evaluated potential electron-tunneling routes across MD snapshots (Onuchic et al. 1992). Because a native PbdA-Pdx complex is unavailable, we aligned PbdA to the P450cam-Pdx complex (PDB: 4JWS) to assign Arg99 as an operational entry reference and the heme propionate as the terminal reference (Supplementary Fig. S9a). It is important to emphasize that the resulting dimensionless *T*_DA_ values act strictly as relative descriptors of MD-sampled geometries and should not be interpreted as validated electron-transfer rates. Trajectory analysis revealed that D242V exhibited slightly broader sampling with intermittent higher-*T*_DA_ states compared to WT and D242S, driven largely by increased geometric variance (Supplementary Figs. 9b-d). While this altered conformational sampling in D242V correlates kinetically with its improved *k*_cat_, this exploratory analysis lacks absolute validation and does not independently prove an electron-transfer gating mechanism. Therefore, we posit that precise structural matching at position 242 primarily enhances turnover efficiency by remodeling the active-site water network (restricting uncoupling), with potential, albeit highly putative, geometric optimizations in the second-sphere coupling route.

### Establishment of a cascade reaction system

**Fig. 6:**
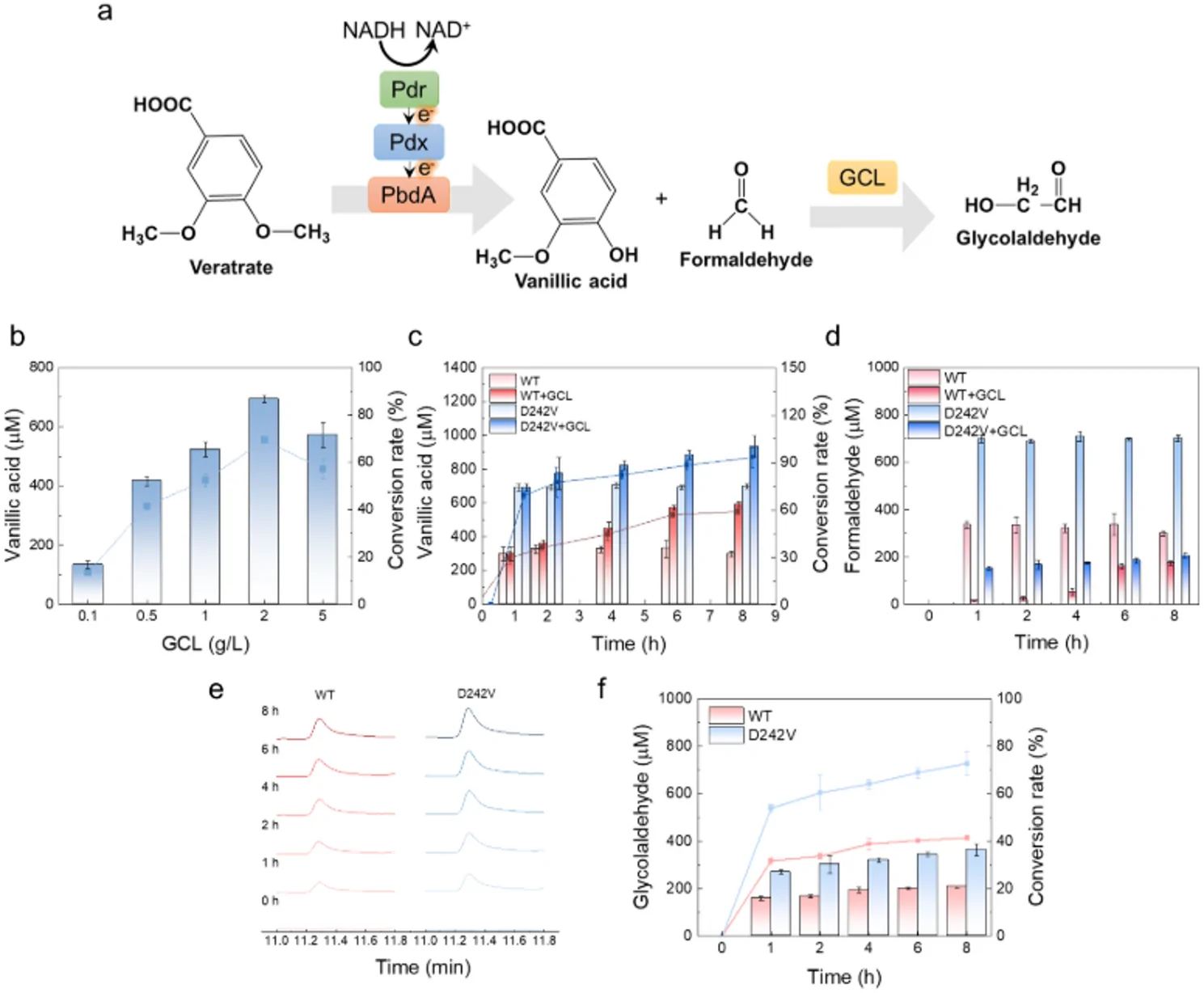
Multi-enzyme cascade for the biotransformation of veratrate and generation of glycolaldehyde. **a** Schematic illustration of the PbdA-GCL cascade system. GCL further catalyzes the carboligation of two molecules of formaldehyde to produce glycolaldehyde (2HCHO→HOCH_2_CHO). **b** Optimization of GCL concentration in the cascade reaction. **c** Time-course analysis of vanillic acid accumulation and substrate conversion catalyzed by WT and D242V with or without optimized GCL-mediated formaldehyde removal over 8 h. **d** Time-course evaluation of formaldehyde generated during the reaction catalyzed by the wild-type (WT) and D242V variant in the presence or absence of the optimized GCL. **e** Representative HPLC chromatograms of GA tracking the reaction progress for the WT (red) and D242V variant (blue) over 8 h. **f** The production of glycolaldehyde and its corresponding conversion rate triggered by the WT or D242V mediated cascade over 8 h. Reaction conditions: 20 mM PBS (pH 7.0), 1 µM P450, 10 µM Pdx, 1 µM Pdr, 1 mM veratrate, 2 mM NADH, 2 g L^− 1^ GCL, 2 mM Mg^2+^, 2 mM TPP and 100 U of catalase. Data are shown as mean ± SD (*n* = 3 independent experiments)

Following identification of D242V and characterization of its improved turnover/coupling, we sought to alleviate the potential inhibitory effects of the toxic byproduct formaldehyde (HCHO). Formaldehyde accumulation may inhibit biocatalysis through nonspecific reactions with proteins and other nucleophiles, potentially contributing to enzyme deactivation or kinetic stalling (Ellis et al. 2021; Calabrese et al. 2026). To this end, a multi-enzyme cascade system was constructed by introducing glyoxylate carboligase (GCL). As illustrated in Fig. 6a, GCL consumes the newly generated HCHO to synthesize glycolaldehyde (GA), a high-value chemical intermediate. GA formation was observed only in the complete formaldehyde/GCL/TPP/Mg²⁺ system (Supplementary Table 3).

To maximize the cascade efficiency, we first optimized the GCL concentration in the reaction mixture (Fig. 6b). The substrate conversion rate peaked when 2 g L^-1^ GCL was added, improving from 58% to over 70% in the initial screening. Higher GCL concentrations (e.g., 5 g L^-1^) resulted in a slight decline in conversion, which may be attributed to molecular crowding or nonspecific protein-protein interactions that interfere with the delicate P450 electron transfer chain (Diamanti et al. 2021; Li et al. 2020). Subsequently, we systematically evaluated the dynamic performance of WT and D242V systems with and without the addition of GCL (Fig. 6c). The improvement is likely attributable to enhanced enzyme or cascade stability resulting from in situ HCHO consumption, rather than to thermodynamic driving of the P450 reaction. As expected, the introduction of GCL boosted conversion in both systems, consistent with a beneficial effect of in situ HCHO clearance on inhibition and enzyme deactivation. Direct HCHO quantification further showed that GCL supplementation reduced HCHO accumulation in both WT and D242V cascade reactions (Fig. 6d). Consistent with its higher intrinsic turnover and improved apparent coupling efficiency, the D242V + GCL system outperformed all other groups, achieving approximately 90% substrate conversion after 8 h, whereas the WT + GCL system reached approximately 60%. Furthermore, HPLC and LC-MS analyses confirmed the robust synthesis of GA over time (Fig. 6e, Supplementary Figs. 10 and 11). However, as shown in Fig. 6f, while substrate conversion reached approximately 90%, the final accumulated yield of GA plateaued at approximately 70% after 8 h.

## Conclusion

This study reveals that predictable second-sphere engineering remains highly challenging. Structure-guided screening around the PbdA-veratrate complex identified D242V as a beneficial substitution at a sensitive second-sphere node. D242V nearly doubled *k*_cat_ and increased apparent coupling efficiency while maintaining WT-like spectral *K*_d_ and apparent *K*_m_ values. In contrast, the hydrophilic D242S substitution impaired catalysis. However, we emphasize a cautious interpretation of these outcomes: the disparity between D242V and D242S alone is insufficient to support the conclusion that generic local hydrophobicity determines catalytic performance. MD simulations suggested that V242 reduces active-site water occupancy and modestly shortens water residence, providing a plausible structural explanation for reduced nonproductive NADH utilization. Pathways analysis revealed altered sampling of putative electron-transfer geometries in D242V, but these computational descriptors should be interpreted as correlative rather than causal. Integration of PbdA with GCL enabled in situ formaldehyde scavenging and glycolaldehyde production, reaching approximately 90% veratrate conversion and 70% GA yield under the tested conditions. Based on the rapid conversion phase during the initial 2 h, this engineered cascade yielded an estimated total turnover number (TTN) of ~ 700 and a space-time yield (STY) of ~ 0.009 g⋅L^− 1^ h^− 1^ for GA. Previous CYP199 enzymes using physiological redox partners have shown higher rates and/or higher substrate loading for veratrate oxidation. Therefore, the present work should not be viewed as surpassing the best CYP199 systems in practical productivity. Rather, it identifies residue 242 in PbdA as a second-sphere control point that modulates active-site hydration and apparent coupling in a heterologous Pdx/Pdr-reconstituted system, and suggests that formaldehyde removal can improve prolonged cascade performance. Therefore, further optimization of the electron-transfer system will be important to improve the overall catalytic efficiency and practical utility of PbdA-based biocatalysis. Overall, while these findings indicate that predictable second-sphere engineering remains highly challenging and context-specific, they do not preclude its potential. The striking divergence between the D242V and D242S variants emphasizes the necessity of exact physicochemical pairing. Consequently, the discovery of D242V provides a targeted blueprint, demonstrating that when precise structural matching is achieved, isolated second-sphere modifications can effectively modulate P450-catalyzed oxidation for biocatalytic applications.

## Supplementary Information

Below is the link to the electronic supplementary material.

Supplementary Material 1.

## Data Availability

All relevant data generated and analyzed during this study are included in this article and its supplementary information. Data are presented as the mean ± standard deviation (SD). Source data are provided with this paper.

## Abbreviations

P450s: Cytochrome P450 monooxygenases
SSM: Site-saturation mutagenesis
Pdx/Pdr: Putidaredoxin/putidaredoxin reductase
CAT: Catalase
MD: Molecular dynamics
RDF: Radial distribution function
*T*_DA_: Dynamic electronic coupling
HCHO: Formaldehyde
GCL: Glyoxylate carboligase
GA: Glycolaldehyde
