# Supplementary material for: A second-sphere variant of cytochrome P450 PbdA improves turnover and apparent coupling in veratrate oxidation
Source: Bioresour Bioprocess. 2026 Sep 10;13(1):131. doi: 10.1186/s40643-026-01130-4 (PMC13562471; doi:10.1186/s40643-026-01130-4)
Supplement: Supplementary file 1 [file 40643_2026_1130_MOESM1_ESM.docx]

**SUPPORTING INFORMATION**

**A second-sphere variant of cytochrome P450 PbdA improves turnover and apparent coupling in veratrate oxidation**

Haiyan Song^1,2^, Zeyang Li^2^, Andong Li^1^, Xinyu Cui^2,3^, Shangxian Xie*^1^, Zhiguang Zhu*^2,3^

1	H Song, A Li, S Xie

Department of Biotechnology, Key Laboratory of Molecular Biophysics of the Ministry of Education, College of Life Science and Technology, Huazhong University of Science and Technology

Wuhan, 430074, China

E-mail: [shangxian_xie@hust.edu.cn](mailto:shangxian_xie@hust.edu.cn)

2	H Song, Z Li, X Cui, Z Zhu

State Key Laboratory of Engineering Biology for Low-carbon Manufacturing, Tianjin Institute of Industrial Biotechnology, Chinese Academy of Sciences

32 West 7th Avenue, Tianjin Airport Economic Area, Tianjin, 300308, China

E-mail: [zhu_zg@tib.cas.cn](mailto:zhu_zg@tib.cas.cn)

3	X Cui, Z Zhu

University of Chinese Academy of Sciences

19A Yuquan Road, Shijingshan District, Beijing, 100049, China

E-mail: [zhu_zg@tib.cas.cn](mailto:zhu_zg@tib.cas.cn)

**Supplementary Fig. 1** Reaction scheme of the regioselective O-demethylation catalyzed by PbdA.

**Supplementary Fig. 2** Standard curve (a) for vanillic acid quantification by HPLC (b).

**Supplementary Fig. 3** Representative HPLC chromatograms of enzymatic reactions catalyzed by the D242V variant with veratrate as substrate. Reactions were quenched at 0 h (black line) and after 0.5 h (red line).

**Supplementary Fig. 4** Re-evaluation of selected variants under optimized conditions. Vanillic acid production (bars, left axis) and conversion rate (line, right axis) of WT and indicated mutants.

**Supplementary Fig. 5** UV-visible absorption spectra monitoring the substrate binding of PbdA WT, D242V and D242S. Darker colors indicate higher concentration of veratrate.

**Supplementary Fig. 6** MD simulation of WT, D242V and D242S. (a) Root-mean-square deviation (RMSD) and (b) root-mean-square fluctuation (RMSF) during 200 ns simulations.

**Supplementary Fig. 7** Hydrophobic desolvation and destabilization of the active site water network in WT, D242V and D242S (two replicates). All analyses were performed on the equilibrated 60 ns trajectory window. (a) Time evolution of the number of water molecules within 10 Å of the heme iron, showing a persistent reduction consistent with the first replicate. (b) Time-dependent analysis of the number of water molecules within 10 Å of the heme center.

**Supplementary Fig. 8** (a) Survival probability of water molecules in the active site, illustrating a faster decay (shorter residence) for the mutant. (b) Probability density distribution of water residence times.

**Supplementary Fig. 9** Structural basis and dynamic electronic coupling (*T*_DA_) analysis of the putative electron-transfer pathway. (a) Structural alignment of the complex structures (PDB 9G9S, pink; PDB 4JWS, purple; RMSD = 1.90 Å), highlighting the proposed coupling‑geometry entry node and terminal heme acceptor. The conserved surface residue Arg99 in 9G9S structurally corresponds to Arg112, a canonical electron-entry portal in 4JWS. Asp38 in 4JWS (implicated in electron delivery from Pdx) and the heme acceptor moieties are indicated. (b) Probability density distributions of the dimensionless coupling metric *T*_DA_ computed from MD snapshots using the VMD Pathways plugin (default parameters), shown as violin plots. Donor and acceptor were defined as the Arg99 CA atom and the heme propionate O2A atom, respectively. (c) Decomposition of the mean-squared coupling <*T*_DA_^2^> into the squared mean <*T*_DA_>^2^ (bottom, purple gradient) and the variance σ^2^ (top, yellow gradient), where <*T*_DA_^2^> =<*T*_DA_>^2^ +σ^2^. (d) Time evolution of *T*_DA_ along the Arg99-to-heme conduit over the analyzed 120-180 ns segment (60 ns). WT (pink) and D242S (orange) fluctuate within a narrow range, whereas D242V (blue) intermittently transient samples snapshots with higher *T*_DA_ scores.

**Supplementary Fig. 10** Standard curve (a) for glycolaldehyde quantification by high-performance liquid chromatography (HPLC) (b).

**Supplementary Fig. 11** LC-MS detection of glycolaldehyde GA. Representative LC-MS chromatogram and mass spectrum showing the GA peak at 8.320 min with the corresponding ion at m/z 222.0697, confirming the detection of GA in the sample.

**Supplementary Table 1.** Primers used in this study.

| Primer | Sequence (5´-3´) |
| --- | --- |
| W81NNK-f | agcaacttccgggacgagaccccgNNKcgtcccccgagtgtcctgctcgag |
| W81NNK-r | ctcgagcaggacactcgggggacgMNNcggggtctcgtcccggaagttgct |
| P84NNK-f | cgggacgagaccccgtggcgtcccNNKagtgtcctgctcgaggccgaccc |
| P84NNK-r | gggtcggcctcgagcaggacactMNNgggacgccacggggtctcgtcccg |
| L87NNK-f | gaccccgtggcgtcccccgagtgtcctgctcgaggccgacccgccgcacc |
| L87NNK-r | ggtgcggcgggtcggcctcgagcaggacactcgggggacgccacggggtc |
| L88NNK-f | gaccccgtggcgtcccccgagtgtcctgNNKgaggccgacccgccgcacc |
| L88NNK-r | ggtgcggcgggtcggcctcMNNcaggacactcgggggacgccacggggtc |
| G169NNK-f | cggacgcgagaacctgctgccgtacnnkgaccacgcattcaacgcgttcg |
| G169NNK-r | cgaacgcgttgaatgcgtggtcmnngtacggcagcaggttctcgcgtccg |
| D171NNK-f | gcgagaacctgctgccgtacggggaccacgcattcaacgcgttcggtccc |
| D171NNK-r | gggaccgaacgcgttgaatgcgtggtccccgtacggcagcaggttctcgc |
| F176NNK-f | ggggaccacgcattcaacgcgnnkggtcccgtcaacagtctggtggagaa |
| F176NNK-r | ttctccaccagactgttgacgggaccmnncgcgttgaatgcgtggtcccc |
| R234NNK-f | gtacgagcaggcgccgctcgtcgtgnnktcgctgctgacggccggggtgg |
| R234NNK-r | ccaccccggccgtcagcagcgamnncacgacgagcggcgcctgctcgtac |
| T238NNK-f | ccgctcgtcgtgcgctcgctgctgnnkgccggggtggacaccacggtcaa |
| T238NNK-r | ttgaccgtggtgtccaccccggcmnncagcagcgagcgcacgacgagcgg |
| A239NNK-f | gcgccgctcgtcgtgcgctcgctgctgacgnnkggggtggacaccacggt |
| A239NNK-r | accgtggtgtccaccccmnncgtcagcagcgagcgcacgacgagcggcgc |
| D242NNK-f | cgctcgctgctgacggccggggtgNNKaccacggtcaacgggctcgcggc |
| D242NNK-r | gccgcgagcccgttgaccgtggtmnncaccccggccgtcagcagcgagcg |
| T243NNK-f | tcgctgctgacggccggggtggacNNKacggtcaacgggctcgcggccgt |
| T243NNK-r | acggccgcgagcccgttgaccgtMNNgtccaccccggccgtcagcagcga |
| V289NNK-f | gtccggtgggagtcgccggtccagacgNNKttccgcaccgccacccgcga |
| V289NNK-r | tcgcgggtggcggtgcggaaMNNcgtctggaccggcgactcccaccggac |

**Supplementary Table 2.** Source organisms, sequences, and accession numbers of the enzymes used in this study.

| Enzyme | Source organisms | Accession number | Sequences |
| --- | --- | --- | --- |
| PbdA | Rhodococcus jostii RHA1 | Q0SCI3 | As shown below |
| Pdx | [Pseudomonas putida](https://www.uniprot.org/taxonomy/303) | [P00259](https://www.uniprot.org/uniprotkb/P00259/entry) |  |
| Pdr | [Pseudomonas putida](https://www.uniprot.org/taxonomy/303) | [M5AXR7](https://www.uniprot.org/uniprotkb/M5AXR7/entry) |  |
| GCL | [Escherichia coli](https://www.uniprot.org/taxonomy/83333) | [P0AEP7](https://www.uniprot.org/uniprotkb/P0AEP7/entry) |  |

The sequences of PbdA, Pdx, Pdr and GCL.

**PbdA WT:**

MGSSHHHHHSSGENLYFQGHMFAPAPALPVSDADPFALDVLQDPLPFQANLRDAGPVVYLRRYDVFALGRYEQVHAALTDWQSFQSAAGVGLSNFRYETPWRPPSILLEADPPHHDAPRAVLSKILGPRALQKLRAAWIQDAEALVDQLLANTTEFDAVTDLAAAFPLRVFPDAVGIPDAGRENLLPYGDHAFNAFGPANGLVEKGAPRVAELSGWVNAQCARDALTGDGFGAQIWAAADRGDITYEQAPLVVRSLLTAGVDTTVNGLAAVLYAFATHPDQWARLRENRTLARTAFDEAVRWESPVQTFFRTATRDTEIGGATIPDGKKILMFLGAANRDPRRWENPEVFDLGRNPSGHVGYGMGIHQCVGQHVARLESEALLTALASRVHSLEIAGPVHRHLNNTLRSWESVPVKVRLP

**Pdx:** MSKVVYVSHDGTRRELDVADGVSLMQAAVSNGIYDIVGDCGGSASCATCHVYVNEAFTDKVPAANEREIGMLECVTAELKPNSRLCCQIIMTPELDGIVVDVPDRQW

**Pdr:** HMNANDNVVIVGTGLAGVEVAFGLRASGWEGNIRLVGDATVIPHHLPPLSKAYLAGKATAESLYLRTPDAYAAQNIQLLGGTQVTAINRDRQQVILSDGRALDYDRLVLATGGRPRPLPVASGAVGKANNFRYLRTLEDAECIRRQLIADNRLVVIGGGYIGLEVAATAIKANMHVTLLDTAARVLERVTAPPVSAFYEHLHREAGVDIRTGTQVCGFEMSTDQQKVTAVLCEDGTRLPADLVIAGIGLIPNCELASAAGLQVDNGIVINEHMQTSDPLIMAVGDCARFHSQLYDRWVRIESVPNALEQARKIAAILCGKVPREEAAPWFWSDQYEIGLKMVGLSEGYDRIIVRGSLAQPDFSVFYLQGDRVLAVDTVNRPVEFNQSKQIITDRLPVEPNLLGDESVPLKEIIAAAKAELSSA

**GCL:**

MAKMRAVDAMYVLEKEGITTAFGVPGGAINPFYSAMRKHGGRHILARHVEGASHMAEGYTRATAGNIGVCLGTSGPAGTDMITALYSASADSIPILCITGQAPRRRLHKEDFQAVDIEAIAKPVSKMAVTVREAALVPRVLQQAFHLMRSGRPGPVLVDLPFDVQVAEIEFDPDMYEPLPVYKPAASRMQIEKAVEMLIQARPVIVAGGGVINADAAALLQQFAELTSVPVIPTLMGWGCRIPDDHELMAGMVGLQTAHRYGNATLLESDMVFGIGNRFAQKHTGSVEKYTEGRKIVHIDIEPTQIGRVLCPDLGIVSDAKAALTLLVEVAQEMQKAGRLPCRKEWVADCQQRKRTLLRKTHFDNVPVKPQRVYEEMNKAFGRDVCYVTTIGLSQIAAAQMLHVFKDRHWINCGQAGPLGWTIPAALGVCAADPKRNVVAISGDFDFQFLIEELAVGAQFNIPYIHVLVNNAYLGMIRQSQMAFDLDYCVQLAFENINSSEVNGYGVDHVKVAEGLGCKAIRVFKPEDIAPAFEQAKALMAQYRVPVVVEVILERVTNISMGSELDNVMEFEDIADNAADAPTETCFMHYE

**Supplementary Table 3.** GCL experimental reaction verification

| Experimental groups | Formaldehyde (1 mM) | GCL (2 g L^-1^) | TPP (2 mM) | Mg^2+^(2 mM) | Glycolaldehyde （mM） |
| --- | --- | --- | --- | --- | --- |
| 1 | + | + | + | + | 0.4 ±0.26 |
| 2 | + | - | + | + | 0 |
| 3 | + | + | - | + | 0 |
| 4 | + | + | + | - | 0 |
| 5 | - | + | + | + | 0 |
